# Presumed hepatic pseudolipoma detected as a small hepatic surface lesion: multimodality imaging findings

**DOI:** 10.1093/bjrcr/uaag040

**Published:** 2026-10-01

**Authors:** Koichi Soga, Ou Takagi, Kohei Inaba, Mikako Masuda, Ikuhiro Kobori, Katsutoshi Sugimoto

**Affiliations:** Department of Gastroenterology, Dokkyo Medical University Saitama Medical Center, Koshigaya, Saitama 343-8555, Japan; Department of Gastroenterology, Dokkyo Medical University Saitama Medical Center, Koshigaya, Saitama 343-8555, Japan; Department of Gastroenterology, Dokkyo Medical University Saitama Medical Center, Koshigaya, Saitama 343-8555, Japan; Department of Gastroenterology, Dokkyo Medical University Saitama Medical Center, Koshigaya, Saitama 343-8555, Japan; Department of Gastroenterology, Dokkyo Medical University Saitama Medical Center, Koshigaya, Saitama 343-8555, Japan; Department of Gastroenterology, Dokkyo Medical University Saitama Medical Center, Koshigaya, Saitama 343-8555, Japan

**Keywords:** hepatic pseudolipoma, hepatic surface lesion, dynamic ultrasonography, computed tomography, magnetic resonance imaging, epiploic appendage

## Abstract

Hepatic pseudolipomas are uncommon benign lesions located on or near the liver capsule which are thought to arise from a detached epiploic appendage that becomes trapped near Glisson’s capsule. Because these lesions may appear as small hepatic or perihepatic nodules, they can mimic primary or metastatic hepatic tumors on screening imaging. Here, we report the case of a 55-year-old asymptomatic man with no relevant medical history who was referred after routine health-screening ultrasonography revealed a 10 × 8-mm hyperechoic lesion adjacent to the surface of liver segment VI. Liver function tests were normal, hepatitis B and C viral markers were negative, and tumor markers, including carcinoembryonic antigen, carbohydrate antigen 19-9, alpha-fetoprotein, and protein induced by vitamin K absence or antagonist-II, were within the normal ranges. Dynamic ultrasonography demonstrated that the lesion was located along the hepatic capsule, rather than within the deep hepatic parenchyma. Computed tomography showed a well-demarcated hepatic surface lesion with central calcification and surrounding fat attenuation, without any arterial, portal venous, or delayed enhancement. Gadoxetate disodium-enhanced magnetic resonance imaging revealed fat-containing signal characteristics, no hepatobiliary-phase uptake, and no diffusion restriction. The integrated multimodal imaging findings supported a diagnosis of presumed hepatic pseudolipoma, likely originating from a detached epiploic appendage. Recognition of this benign hepatic tumor mimic may help to avoid unnecessary biopsy or surgery.

## Introduction

Hepatic pseudolipoma, also known as pseudolipoma of Glisson’s capsule, is a rare, benign capsular or subcapsular lesion composed of degenerated adipose tissue.^1–5^ These lesions are generally considered to originate from a detached epiploic appendage or another mesothelial appendage trapped near the liver capsule and subsequently undergoes degeneration, fibrosis, hyalinization, or calcification.^1^^,^^4–6^ Recent imaging and pathological reports have described noninvasive radiological findings, peritoneal loose-body mimics, migratory behavior, and histologically-confirmed migration from the pelvic cavity to the hepatic surface.^6–9^ Although benign and usually asymptomatic, hepatic pseudolipomas can mimic primary or metastatic liver tumors, particularly when incidentally detected as small nodules on the hepatic surface during abdominal imaging.^2–4^^,^^7^^,^^9^ This diagnostic pitfall is clinically relevant because many such lesions are initially interpreted as small hepatic tumors before their capsular or extrahepatic location is recognized.

With the widespread use of health-screening ultrasonography, asymptomatic hepatic nodules are often detected and referred for further evaluation. In this setting, the key diagnostic challenge is distinguishing true intrahepatic tumors from capsular, subcapsular, or extrahepatic lesions. Dynamic ultrasonography assesses the relationship between a lesion, the hepatic capsule, and adjacent structures in real time, whereas computed tomography (CT) and magnetic resonance imaging (MRI) characterize fat, calcification, enhancement, and diffusion restriction. Therefore, establishing whether a small superficial lesion is intrahepatic or capsular is an important step before invasive diagnostic procedures. However, reports describing the ultrasonographic findings of hepatic pseudolipoma, particularly dynamic B-mode ultrasonographic findings, remain limited.^10^

Herein, we report an asymptomatic case of presumed hepatic pseudolipoma, likely originating from a detached epiploic appendage, initially detected as a small hepatic lesion during health-screening ultrasonography and subsequently diagnosed noninvasively using dynamic ultrasonography, CT, and gadoxetate disodium-enhanced MRI. This case highlights the importance of recognizing this rare mimic of a hepatic tumor during the secondary evaluation following screening ultrasonography.

## Case presentation

### Clinical presentation

A 55-year-old man was referred to our hospital to undergo detailed evaluation of a small hepatic lesion in segment VI detected during routine health-screening ultrasonography. The patient was asymptomatic and had been previously healthy. He had no history of diabetes mellitus, regular medication use, liver disease, malignancy, abdominal surgery, or abdominal trauma. His alcohol intake was occasional, and he had no clinically relevant lifestyle-related disorders.

Physical examination was unremarkable. Laboratory tests revealed no signs of liver dysfunction. Serological tests for hepatitis B and C were negative. Tumor markers, including carcinoembryonic antigen, carbohydrate antigen 19-9, alpha-fetoprotein, and protein induced by vitamin K absence or antagonist II, were within normal ranges. The background liver was considered normal based on the clinical, laboratory, and imaging findings.

### Imaging findings

Abdominal ultrasonography performed during health screening revealed a hyperechoic lesion measuring 10 × 8 mm near the surface of segment VI of the liver (Figure 1). The lesion was small, but conspicuous because of its echogenicity and superficial location. Subsequent dynamic ultrasonography revealed the lesion to be located along the hepatic capsule rather than within the deep hepatic parenchyma. Real-time scanning demonstrated a hepatic surface or capsular location, indicating an extraparenchymal lesion rather than a true intrahepatic tumor (Video S1).

**Figure 1:**
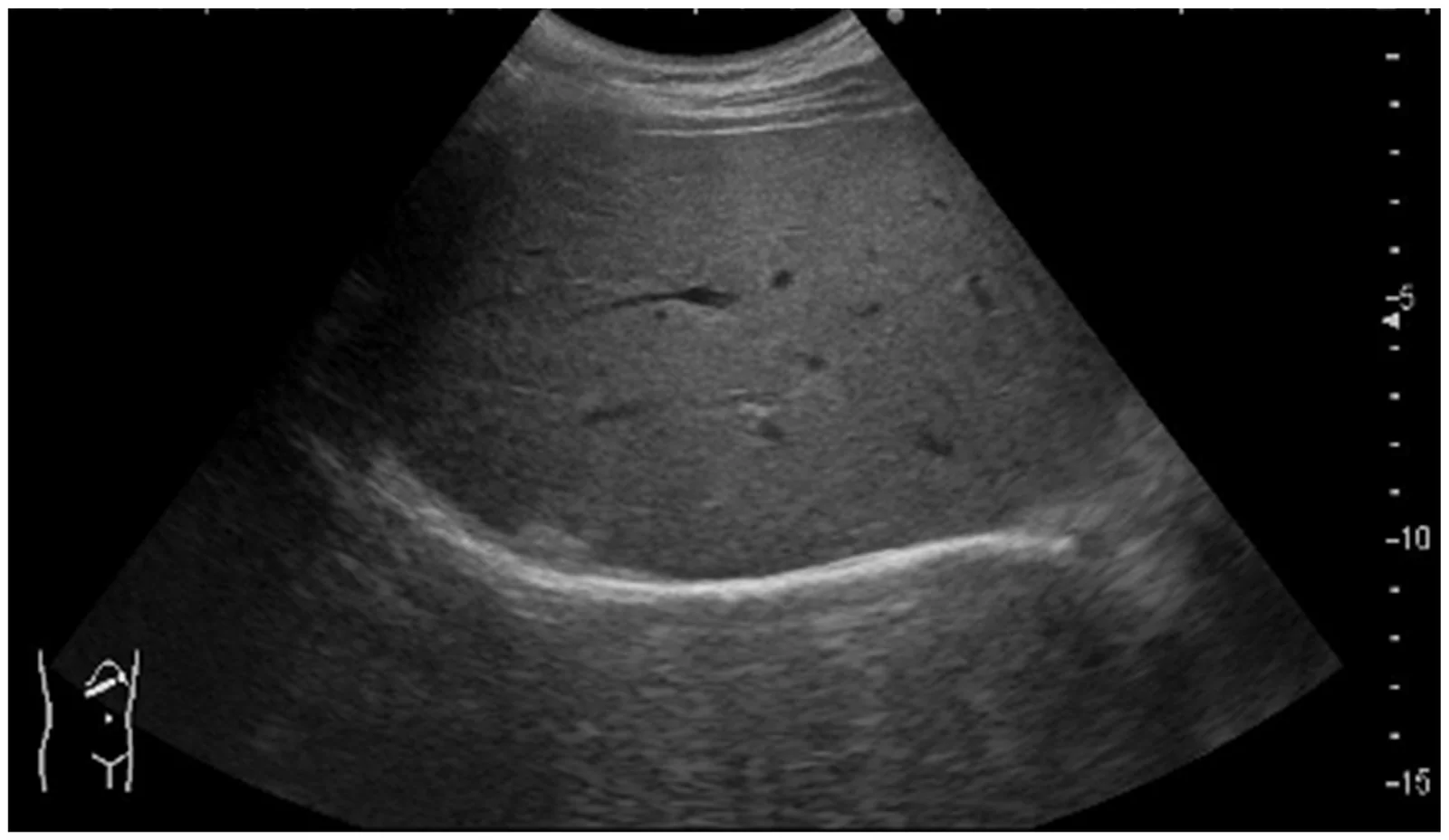
Abdominal ultrasonography performed during health screening. B-mode ultrasonography showed a 10 × 8-mm hyperechoic lesion near the surface of segment VI of the liver. The lesion abutted the hepatic capsule, raising suspicion of a hepatic surface or capsular lesion rather than a true intrahepatic tumor.

Three months after the health-screening ultrasonography, contrast-enhanced CT was performed using a nonionic monomeric iodinated contrast agent. Non-contrast CT demonstrated a relatively well-demarcated, round lesion abutting the segment VI surface. The lesion contained a central high-attenuation focus surrounded by a low-attenuation component, compatible with calcification and fat, respectively. Contrast-enhanced CT revealed no apparent enhancement in the arterial, portal venous, or delayed phases (Figure 2). No other hepatic lesions, abdominal lymphadenopathy, or abdominal findings suggestive of malignancy were observed.

**Figure 2:**
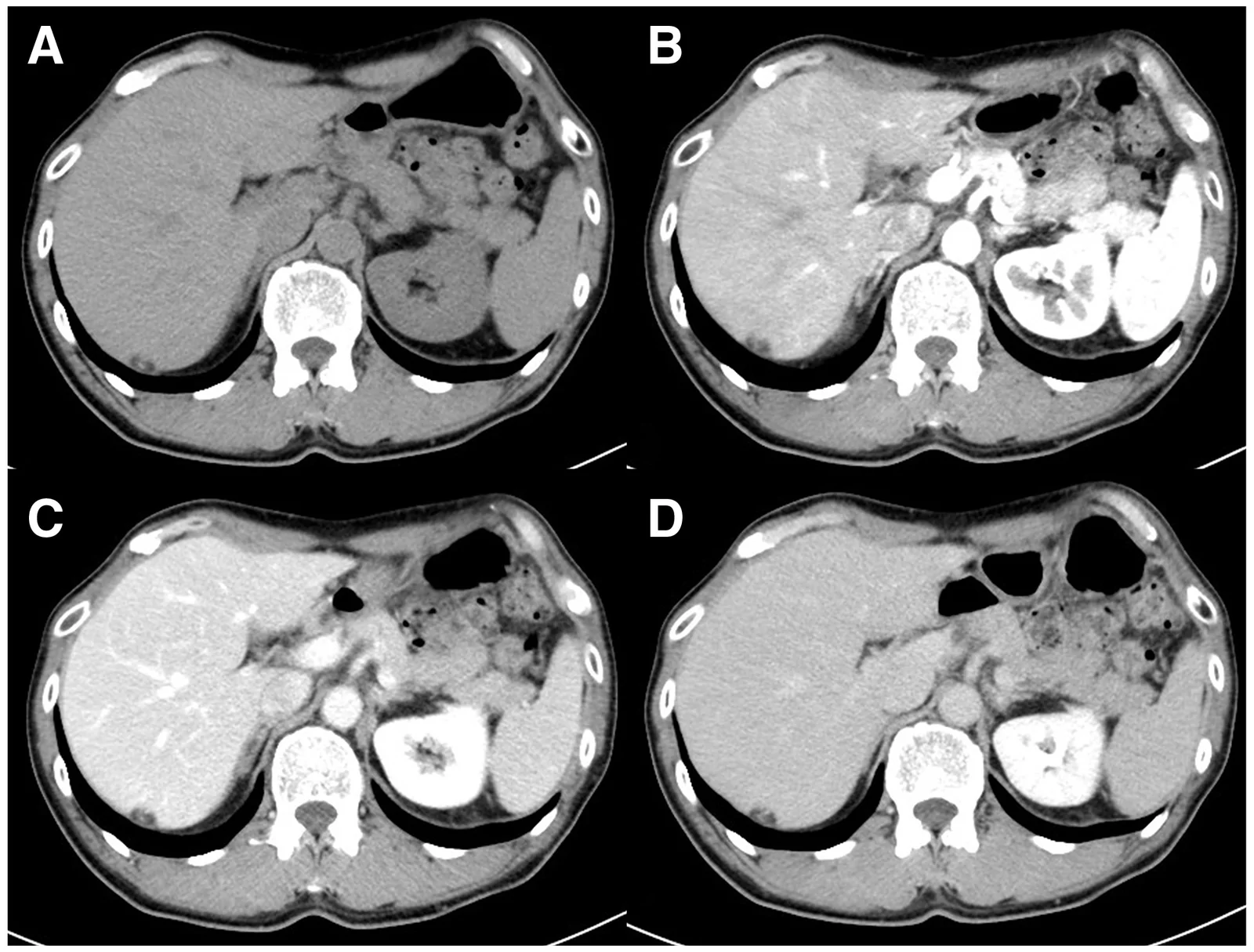
Contrast-enhanced CT of the hepatic surface lesion. Three months after routine health-screening ultrasonography, CT was performed using a nonionic monomeric iodinated contrast agent. Non-contrast CT showed a relatively well-demarcated, small round lesion abutting the surface of segment VI of the liver, comprising a central high-attenuation focus and a surrounding low-attenuation component (A). The central high-attenuation focus was compatible with calcification, whereas the surrounding low-attenuation component suggested fat. Contrast-enhanced CT showed no apparent enhancement of the lesion in the arterial, portal venous, or delayed phases (B-D). These findings supported the diagnosis of a benign fat-containing hepatic surface lesion, most consistent with a presumed hepatic pseudolipoma.

Six months after routine health-screening ultrasonography, MRI was performed using gadoxetate disodium. Hepatobiliary-phase images were obtained 20 min after contrast administration. The T1-weighted mDIXON images revealed the signal characteristics suggestive of a fat-containing component. Axial T2-weighted single-shot imaging confirmed that the lesion was located along the hepatic surface, rather than within the deep hepatic parenchyma. Hepatobiliary-phase gadoxetate disodium-enhanced T1-weighted MRI showed no contrast uptake within the lesion. Diffusion-weighted imaging with a *b*-value of 800 s/mm² and the apparent diffusion coefficient map showed no diffusion restriction (Figure 3).

**Figure 3:**
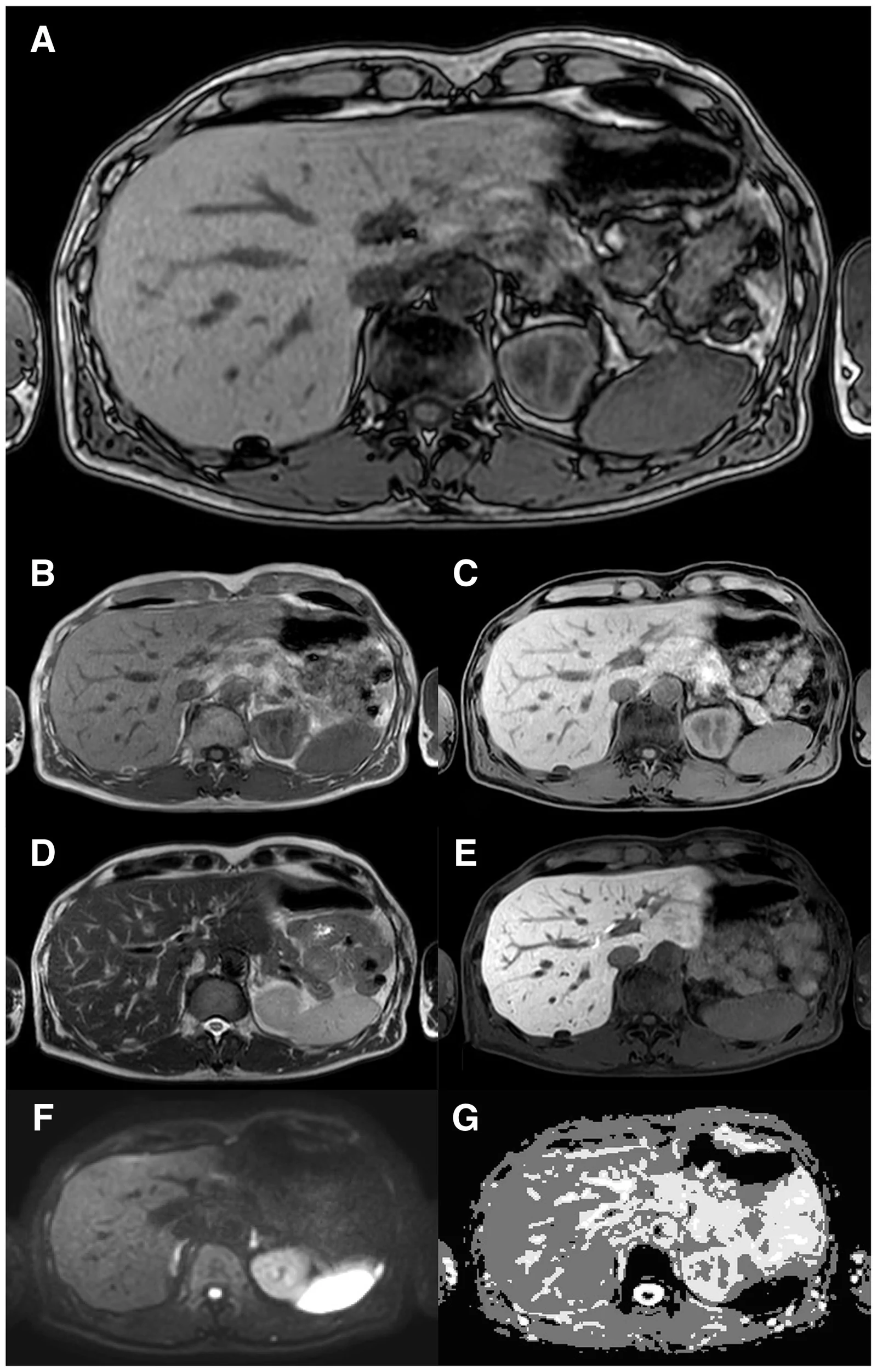
MRI of the hepatic surface lesion. Six months after routine health-screening ultrasonography, MRI was performed using gadoxetate disodium. Hepatobiliary-phase images were obtained 20 min after contrast administration. T1-weighted mDIXON images showed a small lesion abutting the surface of segment VI of the liver, with signal characteristics suggestive of a fat-containing component on opposed-phase, in-phase, and water-only images (A-C). Axial T2-weighted single-shot imaging confirmed that the lesion was located along the hepatic surface rather than within the deep hepatic parenchyma (D). Hepatobiliary-phase gadoxetate disodium-enhanced T1-weighted MRI showed no contrast uptake within the lesion (E). Diffusion-weighted imaging with a *b* value of 800 s/mm² and the apparent diffusion coefficient map showed no diffusion restriction (F, G). These MRI findings further supported the diagnosis of a presumed hepatic pseudolipoma rather than a malignant intrahepatic tumor.

### Differential diagnosis

The main differential diagnoses for the present patient included fat-containing hepatocellular carcinoma, calcified liver metastasis, peritoneal dissemination, hepatic lipoma, angiomyolipoma, solitary necrotic nodule, inflammatory pseudotumor, calcified lymph node, and perihepatic post-surgical/post-inflammatory lesions. Further, because the lesion was small, superficial, fat-containing or calcified, and located on the hepatic surface, hepatic pseudolipoma or a peritoneal loose body attached to the liver surface was also considered.

Based on its hepatic surface location, small size, central calcification, surrounding fat component, lack of enhancement, absence of hepatobiliary-phase uptake, absence of diffusion restriction, normal background liver, negative viral hepatitis markers, normal tumor markers, and lack of clinical findings suggestive of malignancy, the lesion was considered most consistent with a presumed hepatic pseudolipoma, likely originating from a detached epiploic appendage attached to the liver surface. No invasive diagnostic procedures were performed.

### Treatment

Because the imaging findings were largely consistent with a benign hepatic surface lesion, no biopsy, surgical resection, or other invasive diagnostic or therapeutic procedures were performed.

## Outcome, follow-up, and discussion

In the present case, no invasive diagnostic or therapeutic procedures were performed, and the lesion was managed as a presumed benign hepatic surface lesion based on the integrated multimodal imaging findings.

Hepatic pseudolipoma is an uncommon, benign lesion located on or near the liver capsule, ie, histologically characterized by degenerated adipose tissue and may be associated with fibrosis, hyalinization, or calcification.^1^^,^^4^^,^^5^ The most widely accepted mechanism involves the detachment of an epiploic appendage following torsion, ischemia, fat necrosis, and subsequent separation from the colon.^1^^,^^4–6^ The detached appendage may migrate within the peritoneal cavity and become trapped between the liver surface and the diaphragm, or attach to Glisson’s capsule, resulting in a pseudolipomatous lesion.^6^^,^^8^^,^^9^ This concept is supported by early pathological reports and case series of pseudolipoma of Glisson’s capsule,^1^^,^^4^^,^^5^ as well as recent reports describing migration or positional change, including a histologically confirmed case of migration from the pelvic cavity to the hepatic surface.^6^^,^^8^^,^^9^

The clinical importance of hepatic pseudolipomas lies in their potential to mimic hepatic tumors. Previous reports have described cases in which hepatic pseudolipomas or peritoneal loose bodies near the liver were difficult to distinguish from primary or metastatic liver tumors.^2–4^^,^^7^^,^^9^ This diagnostic challenge is particularly relevant when a lesion is detected on screening ultrasonography, as asymptomatic hepatic nodules identified during screening programs often prompt further evaluation. Misinterpretation as a hepatic neoplasm may lead to unnecessary follow-up, biopsy, surgery, or patient anxiety. Therefore, a benign hepatic surface lesion should be considered when a small fat-containing or calcified lesion is found immediately adjacent to the liver capsule.

Previous reports on hepatic pseudolipoma and related hepatic surface lesions are summarized in Table 1. Pathological reports have established hepatic pseudolipoma as a benign capsular lesion, while subsequent imaging reports have described its CT and MRI characteristics, tumor-mimicking behavior, and migratory nature.^1–9^ Contrast-enhanced ultrasonography has been reported to be useful for differentiating hepatic pseudolipoma from metastasis.^10^ In contrast, the present case emphasizes the value of dynamic B-mode ultrasonography in identifying the capsular or hepatic surface location of a small lesion initially detected during health-screening ultrasonography, with subsequent CT and MRI confirmation of benign, fat-containing, and calcified features.

**Table 1:** Overview of representative reports of hepatic pseudolipoma and related hepatic surface lesions.

Reference	Author, year	No. of cases	Diagnostic context	Main modality	Key findings	Pathological confirmation	Relevance to the present case
**1**	Pounder, 1983	1	Early description of hepatic pseudolipoma	Pathology	Degenerated adipose tissue located on or near the hepatic capsule	Yes	Established the pathological concept of hepatic pseudolipoma as a benign fat-related capsular lesion
**2**	Karhunen, 1985	5	Incidental hepatic pseudolipoma with tumor-mimicking potential	Pathology/autopsy	Small capsular or subcapsular fat-related lesions that may be mistaken for primary or metastatic liver tumors	Yes	Highlighted the rarity and clinical pitfall of hepatic pseudolipoma as a liver tumor mimic
**3**	Benbow and Reid, 1986	1	Lesion-simulating metastatic tumor	Pathology/imaging	Pseudolipoma of Glisson’s capsule mimicking metastatic disease	Yes	Supports the need to distinguish this benign capsular lesion from malignancy
**4**	Sasaki et al, 1994	6	Case series and literature review	CT/pathology	CT showed a subcapsular low-density nodule with or without a high-density spot; histology showed a fibrous capsule, necrotic mature fat, and focal calcification in some cases	Yes	Provides important imaging-pathology correlation for small fat-containing or calcified capsular nodules
**5**	Quinn and Guzman-Hartman, 2003	1	Pathological case report	Pathology	Degenerated adipose tissue attached to Glisson’s capsule	Yes	Reinforces the concept that pseudolipoma is a capsular, rather than intraparenchymal, lesion
**6**	Kiolbasa et al, 2021	1	Migrating pseudolipoma	Serial CT	A well-circumscribed hypoattenuating lesion migrated to a hepatic subcapsular location over 4.5 months	No/imaging-based	Supports the hypothesis that a detached epiploic appendage can migrate and become trapped near the hepatic capsule
**7**	Wen et al, 2021	1	Peritoneal loose body presenting as a hepatic mass	CT/MRI/surgery	Perihepatic loose body mimicked a hepatic mass	Yes	Provides an important differential diagnosis for extrahepatic or perihepatic lesions mimicking liver tumors
**8**	Chen et al, 2024	1	Migrating pseudolipoma of Glisson’s capsule	CT/MRI	Migrating fat-containing lesion related to the hepatic capsule	No/imaging-based	Reinforces the importance of serial cross-sectional imaging in diagnosing migratory capsular fat lesions
**9**	Karashima et al, 2025	1	Histologically confirmed migration from the pelvic cavity to the liver	Serial CT, MRI, laparoscopy, pathology	Fat-attenuating pelvic lesions with small calcifications were retrospectively identified; a new well-defined fat-containing lesion later appeared on the hepatic dome; MRI showed no diffusion restriction	Yes	Provides strong pathological evidence that a detached fat-containing peritoneal lesion can migrate to the hepatic surface
**10**	Jiang et al, 2025	1	Ultrasound and contrast-enhanced ultrasound characterization	US/CEUS/CT	Slightly hyperechoic nodule near the liver capsule; no Doppler flow; no enhancement on CEUS; CT showed a fat-density nodule with calcification	No/imaging-based	Highlights the usefulness of ultrasound-based evaluation for differentiating hepatic pseudolipoma from metastasis
Present case	This report	1	Detected during health-screening ultrasonography	Dynamic B-mode US, CT, gadoxetate disodium-enhanced MRI	A 10 × 8-mm hyperechoic hepatic surface lesion; CT showed central calcification and surrounding fat attenuation without enhancement; MRI showed no hepatobiliary-phase uptake or diffusion restriction	No	Demonstrates noninvasive diagnosis after health-screening ultrasonography, with dynamic B-mode US clarifying the hepatic surface or capsular location

Abbreviations: CEUS = contrast-enhanced ultrasonography, CT = computed tomography, MRI = magnetic resonance imaging, US = ultrasonography.

Dynamic ultrasonography was useful because it allowed the real-time assessment of the relationship between the lesion and the hepatic capsule, which was the central diagnostic issue in this case. The lesion appeared as a small hyperechoic mass-like area near the surface of segment VI and was distinct from the deeper hepatic parenchyma during scanning. This finding illustrates an important advantage of ultrasonography because hepatic pseudolipoma is a capsular, subcapsular, or extrahepatic lesion, rather than a tumor arising from the liver parenchyma. Although CT and MRI are useful for tissue characterization, dynamic ultrasonography provides direct anatomical information regarding the surface location of the lesion and may be particularly valuable during the initial assessment after screening ultrasonography, especially when the lesion is small and superficially located.

In the present case, CT and MRI provided complementary diagnostic information; CT revealed a well-demarcated lesion on the hepatic surface with central calcification and surrounding fat attenuation, without enhancement in the arterial, portal venous, or delayed phases. Conversely, gadoxetate disodium-enhanced MRI demonstrated fat-containing signal characteristics, an absence of hepatobiliary-phase contrast uptake, and no diffusion restriction. These findings helped differentiate the lesion from malignant liver tumors, including fat-containing hepatocellular carcinoma, calcified liver metastases, peritoneal dissemination, and other hepatic or perihepatic lesions. The normal liver background, absence of viral hepatitis, normal tumor markers, and lack of imaging features of malignancy further supported a benign diagnosis.

The differential diagnosis also includes hepatic lipoma, angiomyolipoma, solitary necrotic nodule, inflammatory pseudotumor, calcified lymph node, and perihepatic postsurgical or post-inflammatory lesions. In contrast to hepatic pseudolipomas, many of these entities are intraparenchymal, vascularized, associated with a characteristic clinical background, or demonstrate growth, enhancement, or diffusion restriction. In the present case, the combination of a superficial capsular location, central calcification, surrounding fat, absence of contrast enhancement, absence of hepatobiliary-phase uptake, and absence of diffusion restriction was highly suggestive of a benign lesion derived from degenerated adipose tissue, rather than a true intrahepatic neoplasm.

Nevertheless, this report has some limitations. First, the diagnosis was not confirmed pathologically; therefore, the lesion was presumed to be a hepatic pseudolipoma. Second, migration of the lesion from another intra-abdominal site could not be directly demonstrated because no prior imaging was available. Nevertheless, the characteristic multimodal imaging findings strongly supported this diagnosis.

In conclusion, hepatic pseudolipoma should be considered when a small, well-demarcated, fat-containing or calcified lesion is detected on the liver surface during routine health-screening ultrasonography. Dynamic ultrasonography can help distinguish hepatic surface lesions from true intrahepatic tumors. Recognizing this rare benign entity may help prevent unnecessary invasive procedures.

## Learning points

- Hepatic pseudolipoma should be considered for small fat-containing or calcified lesions detected on or near the liver capsule.
- Dynamic ultrasonography can help distinguish hepatic surface lesions from true intrahepatic tumors by demonstrating the relationship of the lesion to the hepatic capsule.
- CT and gadoxetate disodium-enhanced MRI can support a noninvasive diagnosis by showing fat attenuation, calcification, absence of enhancement, absence of hepatobiliary-phase uptake, and absence of diffusion restriction.
- Recognition of this benign hepatic tumor mimic may help to avoid unnecessary biopsy or surgery.

## Supplementary Material

uaag040_Supplementary_Data

## Data Availability

Data sharing is not applicable to this article because no datasets were generated or analyzed.

## References

[1] Pounder DJ. Hepatic pseudolipoma. Pathology. 1983;15:83-84. 10.3109/00313028309061407 6856345

[2] Karhunen PJ. Hepatic pseudolipoma. J Clin Pathol. 1985;38:877-879. 10.1136/jcp.38.8.877 PMC499382 4031100

[3] Benbow EW , Reid H. Pseudolipoma of Glisson’s capsule simulating metastatic tumour. J Clin Pathol. 1986;39:345-346. 10.1136/jcp.39.3.345 PMC499781 3958222

[4] Sasaki M , Harada K, Nakanuma Y, Watanabe K. Pseudolipoma of Glisson’s capsule. Report of six cases and review of the literature. J Clin Gastroenterol. 1994;19:75-78. 7930440

[5] Quinn AM , Guzman-Hartman G. Pseudolipoma of Glisson capsule. Arch Pathol Lab Med. 2003;127:503-504. 10.5858/2003-127-0503-POGC 12683888

[6] Kiolbasa ME , Thimmappa ND, Stensby JD. Migrating pseudolipoma of Glisson’s capsule. Clin Imaging. 2021;69:1-3. 10.1016/j.clinimag.2020.06.033 32645534

[7] Wen Y , Shang MJ, Ma YQ, Fang SH, Chen Y. Peritoneal loose body presenting as a hepatic mass: a case report and review of the literature. Open Med (Wars). 2021;16:1356-1363. 10.1515/med-2021-0351 PMC8435557 34589611

[8] Chen Q , Zhao B, Chen Y, Yang X, Zhou K. Migrating pseudolipoma of Glisson’s capsule: a case report. Radiol Case Rep. 2024;19:5365-5369. 10.1016/j.radcr.2024.08.028 PMC11402135 39280743

[9] Karashima R , Masuda T, Nagayama Y, et al Hepatic pseudolipoma mimicking fat-containing malignant liver tumor: a case of migration from the pelvic cavity. Anticancer Res. 2025;45:4653-4658. 10.21873/anticanres.17811 41006025

[10] Jiang L , Yuan Z, Luo Y. Contrast-enhanced ultrasound findings of hepatic pseudolipoma: a case report. Front Oncol. 2025;15:1581523. 10.3389/fonc.2025.1581523 PMC12353688 40823067

